# ﻿Further taxonomic studies of the mimetic genus *Euryobeidia* Fletcher, 1979 (Lepidoptera, Geometridae, Ennominae, Baptini), with descriptions of four new taxa and two status changes

**DOI:** 10.3897/zookeys.1260.157773

**Published:** 2025-11-21

**Authors:** Bo Liu, Dieter Stüning, Hongxiang Han

**Affiliations:** 1 Coconut Research Institute, Chinese Academy of Tropical Agricultural Sciences, Wenchang, Hainan 571339, China Coconut Research Institute, Chinese Academy of Tropical Agricultural Sciences Wenchang China; 2 Leibniz Institute for the Analysis of Biodiversity Change-Zoological Research Museum Alexander Koenig, Adenauerallee 127, D-53113 Bonn, Germany Leibniz Institute for the Analysis of Biodiversity Change-Zoological Research Museum Alexander Koenig Bonn Germany; 3 State Key Laboratory of Animal Biodiversity Conservation and Integrated Pest Management, Institute of Zoology, Chinese Academy of Sciences, No. 1 Beichen West Road, Chaoyang District, Beijing 100101, China Chinese Academy of Sciences Beijing China

**Keywords:** COI, *
Euryobeidia
*, mimicry, new species, new status, new subspecies

## Abstract

The genus *Euryobeidia* Fletcher, 1979, is reviewed based on morphological characters and available molecular data of cytochrome c oxidase subunit I. Four new taxa of this genus are described: *E. supercostata***sp. nov.** and *E. tigratoides***sp. nov.** from Hainan, China; *E. xuei***sp. nov.** from N. Vietnam and Yunnan, China; and *E. tigratoides leopardiformis***subsp. nov.** from Hubei and Sichuan, China. *E. incrassata* Xiang & Han, 2017 is downgraded to subspecies rank under *E. languidata* (Walker, 1862) (*E. languidata incrassata* Xiang & Han, 2017, **stat. nov.**). The subspecies *E. languidata yakushimensis* Inoue, 1976, is raised to species status (*E. yakushimensis* Inoue, 1976, **stat. nov.**). Adult males and females of all taxa mentioned above, including their genitalia, are illustrated, except for *E. supercostata***sp. nov.** and *E. xuei***sp. nov.**, which are known only from males. An identification key and a geographic distribution map for all known taxa of *Euryobeidia* are presented. The systematics and mimetic relationships of *Euryobeidia* are briefly discussed.

## ﻿Introduction

The genus *Euryobeidia*, belonging to the subfamily Ennominae and, as we know today, to the tribe Baptini, was first completely diagnosed and described by Wehrli (1939: 269) on the basis of two nominal species, *Abraxas languidata* Walker, 1862, from Nepal and *Rhyparia largeteaui* Oberthür, 1884, from “Kouy Tchéou” (Prov. Guizhou, SW. China). Wehrli (1939) separated these from other species of *Obeidia* Walker, 1862 on the basis of their genitalic structures, but, unusually for him, did not designate a type species for his new genus *Euryobeidia* containing the two species in question. Leech (1897: 458) transferred *Rhyparia largeteaui* to *Obeidia* but was uncertain whether this treatment was reasonable and did not make any further comments. Prout (1915: 308) placed *languidata* and *largeteaui* into a separate group of *Obeidia*, based on wing-shape and venation. Many years later, a new taxon related to *E. languidata* from South Japan (Yakushima Island) was described by Inoue (1976: 20) as *E. languidata yakushimensis*, a subspecies with a wing pattern distinctly different from the nominate subspecies. Fletcher (1979: 84, 85) considered *Euryobeidia* Wehrli, 1939 to be not available under section 13(b) of the Code-2 [now article 13.3 of Code-4] and validated the generic name *Euryobeidia* by designating *Abraxas languidata* Walker, 1862 as the type species. By the end of the 20^th^ century, only two species and one subspecies were included in *Euryobeidia* (Parsons et al. 1999: 379). More recently, Sato (2011: 152) considered *E. languidata yakushimensis* to be a distinct species based on its markedly different external characters and differences in the male and female genitalia, compared to the nominate subspecies. This proposal was based on his comparative study of *E. languidata languidata* from the main islands of Japan and *E. languidata yakushimensis* from Yakushima Island, South Japan. However, he hesitated to provide a formal taxonomic treatment and argued that a comprehensive study, including more specimens from outside Japan, was needed to reach a definitive conclusion. Xiang et al. (2017) then reviewed the Chinese species of *Euryobeidia*, describing three new species: *E. ellipsoidea* and *E. quadrata*, both widely distributed, and *E. incrassata*, which is restricted to Hainan Island. They also recorded a few specimens of *E. languidata* from Southeast China and mentioned *E. languidata yakushimensis*, providing a figure of the holotype and a short diagnosis. However, they did not comment or investigate the status of *yakushimensis* by comparing the characters of their new findings to the latter. Rajaei et al. (2022) listed five taxa at the rank of species and two additional taxa at the rank of subspecies within *Euryobeidia*. To date, no additional *Euryobeidia* taxa have been recorded from their natural distribution range in East, Southeast, and South Asia.

In this paper, the genus *Euryobeidia* Fletcher is reviewed, and four new taxa of *Euryobeidia* from China and Vietnam are described and illustrated. Additionally, we propose new status designations for *E. incrassata* Xiang & Han, 2017, and *E. languidata yakushimensis* Inoue, 1976.

## ﻿Material and methods

### ﻿Acronyms and collections

**CKY** Collection of Katsumi Yazaki, Tokyo, Japan;

**CRICATAS** Coconut Research Institute, Chinese Academy of Tropical Agricultural Sciences, Wenchang, China;

**CRS** Collection of Rikio Sato, Niigata, Japan;

**IZCAS** Institute of Zoology, Chinese Academy of Sciences, Beijing, China;

**KIZCAS** Kunming Institute of Zoology, Chinese Academy of Sciences, Kunming, China;

**NHMUK** Natural History Museum, London, United Kingdom;

**NIAES** Institute for Agro-Environmental Sciences, NARO, Tsukuba, Japan;

**ZFMK** Zoologisches Forschungsmuseum Alexander Koenig, Bonn, Germany;

**ZSM** Zoologische Staatssammlung München, Germany.

### ﻿Morphology

Terminology for wing venation follows the Comstock-Needham System (Comstock 1918) as adopted for Geometridae by Scoble (1992) and Hausmann (2001), and that of the genitalia was based on Klots (1970) and Skou and Sihvonen (2015). Abdomens were completely removed and briefly immersed in a boiling 10% KOH solution for maceration of the genitalia, which were then dissected in a 10% ethanol solution and stained with Chlorazol Black E. Photographs of adults and genitalia were taken with digital cameras, those of the genitalia by attaching the camera to a microscope.

### ﻿DNA barcoding

This study used both newly generated sequences and publicly available data. Two sequences were downloaded from the Barcode of Life Data Systems (BOLD: Ratnasingham and Hebert 2007), while the remaining sequences were newly obtained during this study and deposited in GenBank. Detailed sampling data for molecular analyses are presented in Table 1. For new sequences, genomic DNA was extracted from the legs of dried adult specimens. Sanger sequencing was then performed on fresh specimens using the primer pairs: LCO-1490 and HCO-2198, or LepF1 and LepR1 (Folmer et al. 1994; Hebert et al. 2004). Only a small number of older museum specimens were subjected to next-generation sequencing. The molecular analysis in this study included most known *Euryobeidia* taxa, with the exception of *E. yakushimensis* and *E. supercostata*. Material for *E. yakushimensis* was unavailable, and the single known *E. supercostata* specimen failed sequencing due to contamination. A neighbor-joining tree was constructed based on the Kimura 2-parameter method using MEGA 12 (Saitou and Nei 1987; Kimura 1980; Kumar et al. 2024). Genetic distances within and among species are reported as uncorrected pairwise distances (p-distance).

**Table 1.** Sampling data used for molecular analyses in this study.

Voucher/Sample ID	Taxa	locality	GenBank accession numbers/BOLD Process ID
CRICATAS00283	*Euryobeidia languidata languidata*	Fujian, China	PQ083540
CRICATAS00220	*Euryobeidia languidata incrassata*	Hainan, China	PQ083537
CRICATAS00222	*Euryobeidia languidata incrassata*	Hainan, China	PQ083539
ARB00027908	*Euryobeidia xuei*	Yunnan, China	QMA5626-13
IOZ LEP M 52355	*Euryobeidia ellipsoidea*	Gansu, China	PX525429
CRICATAS00247	*Euryobeidia tigratoides tigratoides*	Hainan, China	PQ083534
CRICATAS00239	*Euryobeidia tigratoides tigratoides*	Hainan, China	PQ083538
IOZ LEP M 22920	*Euryobeidia tigratoides leopardiformis*	Sichuan, China	PX525430
CRICATAS00276	*Euryobeidia largeteaui*	Hunan, China	PQ083536
CRICATAS00263	*Euryobeidia largeteaui*	Hunan, China	PQ083541
CRICATAS00250	*Euryobeidia quadrata*	Hunan, China	PQ083535
CCDB-11874-H01	*Eurychoria flavirupta*	Papua New Guinea	PNGTY180-12
CRICATAS00211	*Epobeidia tigrata*	Hainan, China	PQ083279

Note: All the sequences generated in this study are available in the Suppl. material 1.

## ﻿Results

### ﻿Taxonomic account

#### ﻿ Euryobeidia

Taxon classificationAnimaliaLepidopteraGeometridae

Fletcher, 1979

06859937-768E-5FF6-AE5E-640871AC9B9E

Euryobeidia Wehrli, 1939, in Seitz, Gross-Schmett. Erde 4 (Suppl.): 269. Unavailable, type species not designated.
Euryobeidia Fletcher, 1979, The Generic Names of Moths of the World, 3: 84. Type species: Abraxas languidata Walker, 1862.

##### Generic description.

**Figure 1.**
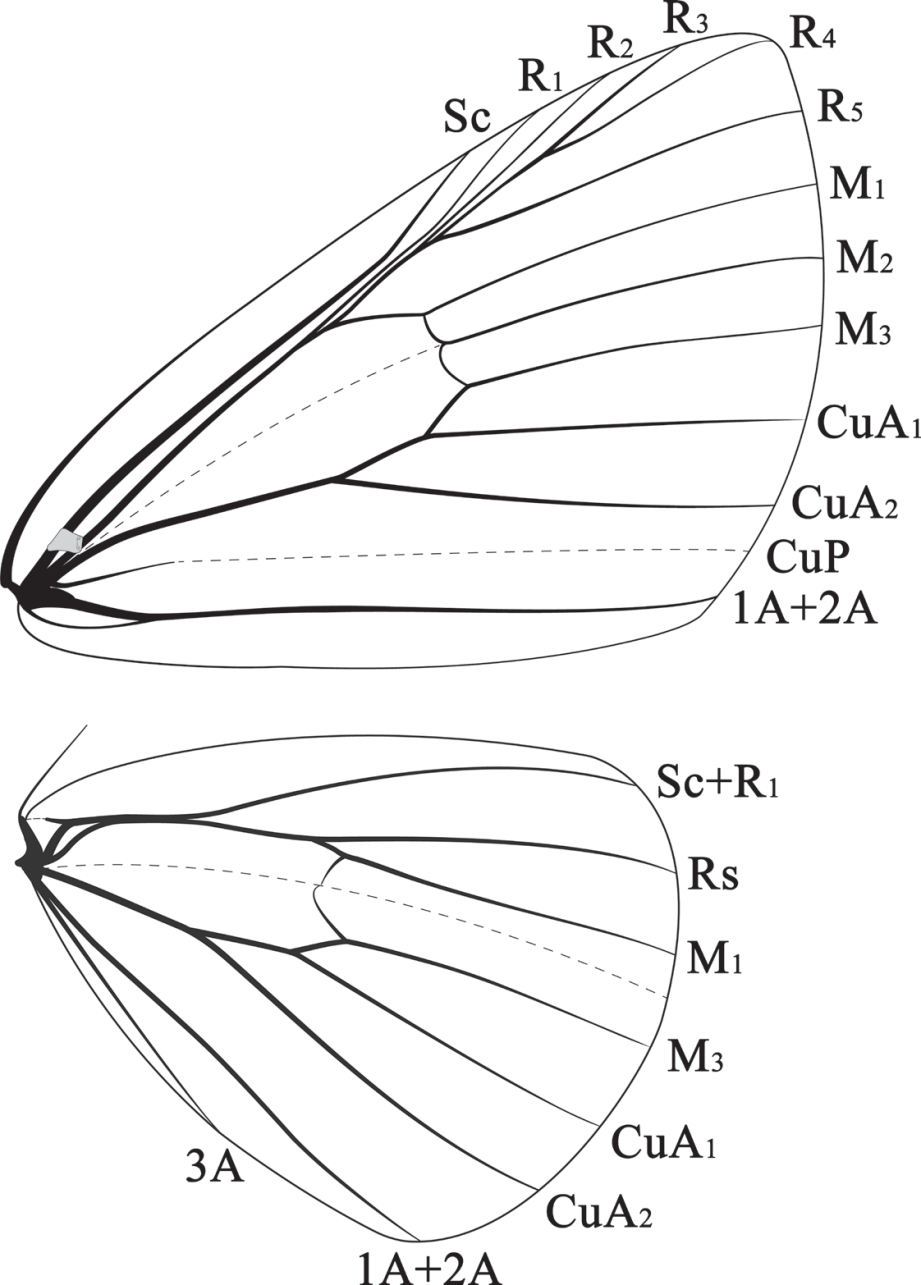
Wing venation of *Euryobeidia tigratoides* sp. nov.

***General appearance*.** Medium-sized ennomine moths, forewing length 17–27 mm. Included species are separated into the following two groups based on ground color: 1^st^ group, including the type-species *E. languidata*, with ground color white to grayish-white; 2^nd^ group orange, often fading to yellow over time. Wings with a large number of black, dark gray or dark brown spots, arranged in a similar pattern, with the exception of *Euryobeidia yakushimensis* stat. nov. ***Head*.** Antennae filiform in both sexes, flagellomeres of a short proximal part cylindrical, more distally they are laterally flattened, ventrally elongated, wedge-shaped, homogeneously covered with very short setae and with a pair of long, straight, spine-like setae, arising mid-laterally on either side near distal margin of each segment. Antennae of females similar, but thinner. Frons narrow, covered with slightly elongated or almost hair-like scales, the latter basally arranged around a tiny, central protrusion of the head-capsule; a concentric arrangement of scales on top of frons, between the bases of antennae, has been observed in some specimens of different species. Vertex covered with slightly longer and broader, distally dentate, obliquely erect scales. Labial palpi slender, roundly curved upwards, just reaching or protruding slightly beyond the frons, third joint small, but clearly visible. Proboscis rather short. Chaetosemata small, near eye margin. ***Thorax*.** Dorsum orange, yellow or grayish-yellow, typically with black dots: one on each patagium, two on each tegula, and two on mesothorax; slight variation occurs among species or individuals. Patagia and tegulae with lamellar, partly elongated scales, tegulae in addition with long hair-scales. Legs slender, pale yellow or orange, with a few dark gray or black dots. Index of spurs 0-2-4, hind tibia not dilated and without scent-brush (hair-pencil) in males. Forewing not or slightly elongate, arched at basal part of costa, apex angled, termen smoothly curved. Fovea absent. Hindwing with a large white area at the basal ⅔–¾ (except for *E. tigratoides tigratoides* and *E. yakushimensis*), and a broad, rarely narrow, yellow, submarginal band with multiple black streaks or dots. Marginal line with black dots at vein-ends, absent in forewing of 1^st^ group, absent or strongly reduced in species of 2^nd^ group. Apex of hindwing rounded, termen minutely concave between vein-ends, posterior margin slightly truncated from the end of vein 3A to tornus and also on termen from vein-end of CuA_2_ to tornus. ***Venation*** (Fig. 1). Forewing: costal area very broad at basal ⅔; Sc evenly curved, but rather abruptly bent upwards near the distal 1/5 (most clearly noticeable in *E. tigratoides*, see Fig. 1); R_1_ arising from upper vein of cell rather close to the common stem of R_2-5_; R_1_ also often exhibits a distinct curvature opposite to the basal ends of R_2_ and R_5_ and a second curvature more distally and therefore reaches the costa closer to the apex; R_2_, as a typical character for the tribe Baptini, arises from the common stem of R_3-5_; stem R_2-5_ arising on a rather large distance from anterior angle of cell (origin of M_1_); M_2_ from the middle of the discocellulars; CuA_1_ widely separated from posterior angle of cell; CuP represented by a rather distinct fold, but with a very short and weak sclerotized vein near the base. Hindwing: Sc+R_1_ running closely parallel but not anastomosing with upper vein of cell at base (a common character of most ennomine moths); Rs arising at a rather small distance from anterior angle of cell; M_2_ absent; CuA_1_ arising at a longer distance from posterior angle of cell; 3A present. ***Pregenital abdomen*.** Abdomen densely covered with fine, whitish, orange, or pale yellow scales. Dorsal side usually with dark spots or transverse bands on each segment from T1 to T8, the spots vary in coloration and size between species and also individually, and rarely may even be absent in some or all segments (e.g. *Euryobeidia tigratoides tigratoides*). Laterally, a row of dark spots present, reduced or absent. Ventral side often with a small number of dark spots and patches of various size and shape, especially in the species-group with white or grayish-white ground color, fewer or even absent in the group of orange/yellow specimens. Integument with most tergites and sternites not conspicuously modified. T1 narrower than T2, appendages of intersegmental tergal phragma T1/T2 very long, strap-like. The 8^th^ segment in males slightly elongate, broader than the 7^th^ segment, posteriorly slightly sclerotized. In females, the 7^th^ segment distinctly enlarged, 8^th^ segment very small, tergite T8 rather membranous, with a cup-shaped, round or oval invagination of unknown function (See the black arrow on Fig. 50; visible on dorsal side, behind the right arm of lamella postvaginalis; so far only observed in *E. languidata*). Tympanal organs of moderate size, shallow, without lacinia. Setal comb on third sternite and sterno-tympanal process both absent, which is consistent with the absence of dilated hind-tibiae with scent-brushes. Coremata not developed.

***Male genitalia*.** Very heterogeneous, especially the uncus with various peculiar shapes among species. It may exhibit the shape of a bird’s head, densely covered with setae, with a small or larger beak-shaped apex, pointed in lateral view, and a narrow neck of differing length. In addition, large, plate-like appendages from “head” may be present. The base of the uncus mostly is a transverse straight or triangular bar, with lateral socii, covered with fine setae. Only one species (*E. largeteaui*) exhibits a rather unmodified uncus. Common characters are rare, e.g. the weak gnathos, reduced to fine, sclerotized lateral arms, not fused at middle, or the large tegumen, consisting of two narrow, elongate arms, which are rather common also in other genera of Baptini. In *Euryobeidia*, the tegumen sometimes is swollen distally and curved ventrad, in other species it is not swollen, but stronger distally and narrower caudally. Transtilla sinuous, broad, band-shaped, slightly to heavily sclerotized. Vinculum short and strong, fused laterally with tegumen. Saccus short, rounded. Juxta groove-like. Valva long and slender, with a well-developed cucullus, reaching from apex back to center of valva, ending with a small, setose knob. Valva distally strongly curved dorsad, apex with a smaller or larger, sometimes lobe-like protrusion, resulting in a more ventral notch. Costa smooth or slightly protruding or with a large, broad process. Base of sacculus often with a lamellar, oval or triangular projection, dorsally more or less dentate and variable in size among species. Aedeagus elongate, apically narrowed, and sclerotized ventrally, often with a pointed process of variable length (extremely long in *E. largeteaui*). Cornuti spine-like or replaced by sclerotized folds and patches of vesica of various shapes. Bulbus ejaculatorius shorter than the aedeagus shaft; proximal part (near aedeagus) tube-like and short; central part U-shaped, open ventrally; distal part cap-shaped, large, with a long rectangular extension distally (Figs 40, 45, 46, 49).

***Female genitalia*.** Ovipositor short, papillae anales slightly elongated, densely and shortly setose, tip rounded. Posterior apophyses long and narrow, anterior apophyses shorter, strong, ~⅓–½ the length of posterior apophyses, their bases dilated or narrow. A triangular sclerite present between the bases of posterior apophyses, its size and shape vary considerably between species and subspecies. Sterigma well developed, sclerotized; lamella antevaginalis spined on posterior margin and more strongly so laterally; lamella postvaginalis a sclerotized plate of different shape, consisting of two layers of plates, connected proximally. Introitus bursae strongly sclerotized, more or less twisted, usually placed asymmetrically on left side in ventral view (situated in the center in *E. largeteaui* only). Ductus seminalis arising close to the end of the short, narrow, strongly sclerotized posterior part of bursa copulatrix (ductus bursae of authors). Anterior part of bursa large, oval or pyriform, distal ½–¾ abundantly spined inside, remaining proximal portion membranous, without spines.

##### Distribution.

China, Korea, Japan, India, Nepal, Vietnam (new record).

##### Diagnosis.

*Euryobeidia* species are very similar in appearance to certain species and subspecies of the *Obeidia*-complex (Inoue, 2003) of genera (mainly of the genus *Epobeidia* Wehrli, 1939) and the genus *Abraxas* Leach, [1815], which probably serve as models in a mimicry relationship. However, they are easily distinguished from the previously mentioned genera by their less elongate, basally arched forewings and markedly different genitalic structures. Within the tribe Baptini, *Euryobeidia* species can be easily identified by the pattern of prominent dark spots on the wings and the distinctive genitalic structures, particularly the peculiar uncus of the male genitalia.

### ﻿Key to all known species of *Euryobeidia* based on characters of external appearance and male genitalia

**Table:** 

1	Ground color of wings white or grayish-white	**2**
–	Ground color of wings orange or yellow (when faded)	**7**
2	Costa of valva in male genitalia smooth, narrow	**3**
–	Costa of male genitalia dilated	**4**
3	Smaller in average size; color of gray-black pattern elements deep when fresh; yellow marginal band on hindwing broad (Figs 2–8, 56)	***E. languidata languidata***
–	Larger in average size; color of gray-black pattern elements much paler when fresh; yellow marginal band on hindwing narrow (Figs 10–13, 57)	***E. languidata incrassata* stat. nov.**
4	Costa of valva in male genitalia significantly large and broad (Fig. 44)	***E. supercostata* sp. nov.**
–	Costa of valva in male genitalia only slightly dilated	**5**
5	Uncus apically strongly modified, with a large plate on a short stem; wing pattern featuring the typical dark spots	**6**
–	Uncus apically not or only slightly dilated, without a plate-like process; wing pattern without dark spots, except on hindwing margin (Figs 16, 17, 42, 43)	***E. yakushimensis* stat. nov.**
6	White area on hindwing smaller and yellow distal band broader; apex of valva strongly curved, terminal lobe of costa large; stalk supporting the large dorsal plate of uncus very short (Figs 9, 18; figures of genitalia see Xiang et al. 2017)	***E. ellipsoidea***
–	White area on hindwing larger and yellow distal band narrower (Fig. 19); apex of valva more strongly curved, terminal lobe of costa much larger (see dotted circle on Fig. 45); stalk supporting the large dorsal plate of uncus long and narrow, shape of plate different	***E. xuei* sp. nov.**
7	Basal process of sacculus broad; apical lobe and neighboring notch of valva conspicuous	**8**
–	Basal process of sacculus narrow; apical lobe and neighboring notch of valva inconspicuous or absent	**9**
8	Larger in average size; hindwing entirely orange, without white basal area; uncus slightly shorter, with the stem strongly curved (Figs 32–37, 46, 47)	***E. tigratoides* sp. nov.**
–	Smaller in average size; hindwing with a large white basal area; uncus slightly longer, with the stem straight or only slightly curved (Figs 27–31, 48, 49)	***E. tigratoides leopardiformis* subsp. nov.**
9	Aedeagus short, apex broad and truncate; uncus irregularly shaped, with a large, quadrate plate posteriorly; hindwing with a white basal area, in addition, forewing with a white, but often indistinct area near hind margin, also present on underside (see Figs 25, 26; figures of genitalia see Xiang et al. 2017: figs 19, 24, 29)	***E. quadrata***
–	Aedeagus long, with a long, hook-like extension apically; uncus elongate, semi-circularly extended on dorsal side, densely setose, without further modifications; forewing without white or with traces of white in a few individuals only, but always present on underside (see Figs 20–24; figures of genitalia see Xiang et al. 2017: figs 18, 23, 28)	***E. largeteaui***

#### ﻿ Euryobeidia languidata

Taxon classificationAnimaliaLepidopteraGeometridae

(Walker, 1862)

4423D5E6-8DAA-5785-B2D6-1D86ED21263B

Figs 2–8, 38, 39, 50, 56

Abraxas languidata Walker, 1862, List Specimens lepid. Insects Colln Br. Mus. 24: 1122. Holotype, Nepal. (NHMUK)
Euryobeidia languidata : Wehrli 1939, in Seitz, Gross-Schmett. Erde 4 (Suppl.): 269. Unavailable.
Euryobeidia languidata : Fletcher 1979, The Generic Names of Moths of the World 3: 84; Stüning, D. 2000, Moths of Nepal, part 6: 110; Xiang et al. 2017, Zootaxa 4317 (2): 371.

##### Type material examined.

**Figures 2–19.**
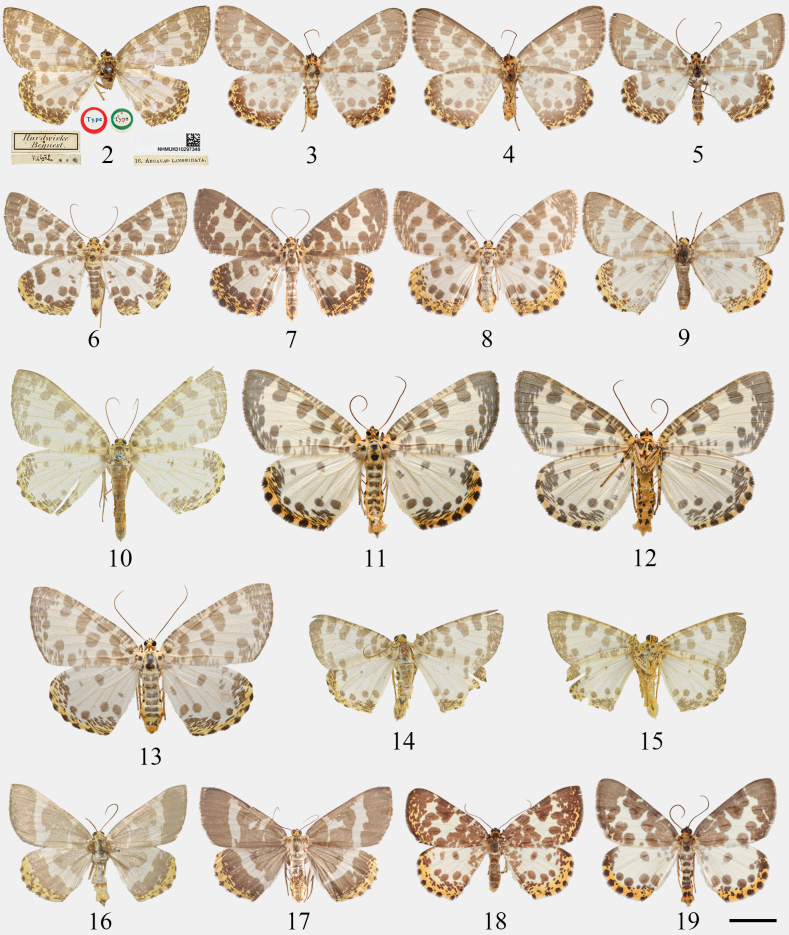
Adults of *Euryobeidia* species. **2–8.***E. languidata* (Walker): **2.** The holotype of *Abraxas languidata*, female, Nepal, NHMUK; **3.** Female, Nepal, upperside, ZSM; **4.** Ditto, underside; **5.** Male, Khasi Hills, India, ZFMK; **6.** Male, N. Vietnam, CKY; **7.** Male, Tokyo, Japan, CRS/ NIAES; **8.** Female, Niigata, Japan, CRS/ NIAES; **9.***E. ellipsoidea*, untypical pattern, female, Sichuan, China, ZFMK; **10–13.***E. languidata incrassata* Xiang & Han, stat. nov., Hainan Is., China: **10.** The holotype of *E. incrassata* Xiang & Han, male, IZCAS; **11.** Non-type, male, upperside, CRICATAS; **12.** Ditto, underside; **13.** Non-type, female, CRICATAS; **14, 15.***E. supercostata* sp. nov., holotype, male, Hainan, China, IZCAS: **14.** Upperside; **15.** Underside; **16, 17.***E. yakushimensis* Inoue, stat. nov., Yakushima Is., Japan: **16.** The holotype of *E. languidata yakushimensis* Inoue, male, NHMUK; **17.** Non-type, female, CRS/ NIAES; **18.***E. ellipsoidea*, paratype, female, Sichuan, China, ZFMK; **19.***E. xuei* sp. nov., paratype, male, Fansipan, Vietnam, ZFMK. Scale bar: 10 mm.

***Holotype*.** Nepal • ♀; ‘Nepal’; Hardwicke Bequest; NHMUK. (Abdomen lost; see Fig. 2)

##### Additional material examined.

Nepal • 1 ♀; Kathmandu Valley, Godavari; 1600–1800 m; 5 Jun.1967; leg. Dierl-Schacht; ZSM. India • 1 ♂; Khasis; May 1896; Nat. Coll.; coll. Wehrli, ZFMK. China – **Fujian Province** • 1 ♂; Quanzhou City, Dehua County, Jiuxianshan; 1200 m; 12 May 2024; C. L. Huang & Z. Peng leg.; GenBank no.: PQ083540; gen. prep. no. CRICATAS00283; CRICATAS – **Taiwan** • 1 ♂; Nantou, Sang-Gan nr. Pu-li; 23°58'N, 120°55'E; Apr.–Jul. 2008; local coll.; ZFMK. Vietnam • 1 ♂; N. Vietnam, Vinhu, Tam Dao; 900 m; 29 May 1997; B. Tanaka leg.; CKY. Japan – • 1 ♂, 1 ♀; ‘Japon, Wileman, 1898’; male gen. prep. Wehrli no. 5525, female gen. prep. no. 2460-DS; ZFMK – **Tokyo** • 1 ♂; Tokio; 17 Jun. 1910; gen. prep. Wehrli. no. 5517; ZFMK • 1 ♂; Abiko, Chiba Pref. nr. Tokyo; May 1914; ZFMK • 1 ♂; Chiyoda, Kokyo; 21 May 1998; Y. Kishida leg.; gen. prep. no. RS8877; CRS/ NIAES (shown by Sato 2011, pl. 1-015-9) • 1 ♀; Chiyoda, Kokyo; 11 Jun. 1998; Y. Kishida leg.; gen. prep. no. RS8878; CRS/ NIAES (shown by Sato 2011, pl. 1-015-10) – **Niigata Prefecture** • 1 ♀; Niigata City, Nishikan-ku, Iwamuro, Parking of Yahiko; 23 Jul. 2007; R. Sato leg.; CRS/ NIAES (shown by Sato 2011, pl. 1-015-11).

##### Remarks.

*Euryobeidia languidata* obviously is extremely rare at its type locality, Nepal. The female holotype at NHMUK is the only specimen among a collection of ~50 specimens, half of them originating from NE India, Assam-Meghalaya region, the second half from Japan. The volumes 1–5 of the “Moths of Nepal”-series do not mention it and several thousand specimens from many localities in Nepal at ZFMK definitely do not contain it. The doubt about the correctness of the type locality has arisen, but was answered by the fact that a second specimen was found at the ZSM collection, Munich, a female collected near Kathmandu, the specimen mentioned above (Stüning 2000: 110, Moths of Nepal, vol. 6). Unfortunately, this and a male from Khasi Hills, Meghalaya could be studied by images only (Figs 2–5). However, in our experience they will not affect our taxonomic conclusions when investigated more closely in future.

##### Diagnosis.

Images of adults and genitalia of the nominate subspecies are shown here for comparison with *E. languidata incrassata* stat. nov. and *E. yakushimensis* stat. nov. The diagnoses are given under each of the two taxa.

##### Distribution.

Nepal (type-locality), India (Assam-Meghalaya), N. Vietnam, China (Sichuan, Yunnan, Jiangxi, Zhejiang, Fujian, Taiwan, Guangdong, Guangxi), Korea, Japan.

##### Remarks.

*Euryobeidia languidata* is the type-species of *Euryobeidia*, designated by Fletcher (1979), and a member of the white or grayish-white group of species (1^st^ Group). It is the species with the widest area of distribution, from east Nepal in the west to north Japan in the east, with southernmost occurrence on the island of Hainan (ssp. incrassata). Within China, it appears to be primarily distributed along the southeastern coast. More to the north, there appears to be a gap between northern Zhejiang and South Korea, but this region is mostly low-lying plains with high population density, extensive agriculture and hardly any forests, obviously unsuitable for the existence of this species.

#### ﻿ Euryobeidia languidata incrassata

Taxon classificationAnimaliaLepidopteraGeometridae

Xiang & Han stat. nov.

0899FC5C-5BAF-53D6-97B6-73F7883E65C9

Figs 10–13, 40, 41, 53, 57

Euryobeidia incrassata Xiang & Han, 2017, Zootaxa 4317 (2): 372 (part).

##### Type material examined.

***Holotype*.** China – **Hainan Province** • ♂; Lingshui, Diaoluoshan; 8 May 1984; coll. Gu Maobin; IOZ-CAS slide no. Geom-7235; IZCAS. ***Paratypes*.** China – **Hainan Province** • 1 ♂; same collection data as for holotype; IZCAS • 1 ♂; Baisha, Nankai, Nanmaola; 1261 m; 12–14 May 2009; coll. Chen Fuqiang; IZCAS.

##### Additional material examined.

China – **Hainan Province** • 3 ♂♂, 1 ♀; Lingshui, Diaoluoshan; 922 m; 10 May 2023; Bo Liu leg.; GenBank no.: PQ083537; gen. prep. nos. CRICATAS00220, CRICATAS00223; CRICATAS/ ZFMK • 1 ♂; same locality as for preceding; 07–12 May 2024; Bo Liu & Wei Yan leg.; GenBank no.: PQ083539; gen. prep. no. CRICATAS00222; CRICATAS • 1 ♂; Lingshui, Diaoluoshan; 997 m; 21 Apr. 2025; Bo Liu & Wei Yan leg.; CRICATAS.

##### Diagnosis.

*Euryobeidia languidata incrassata* Xiang & Han, 2017 differs significantly from the nominate subspecies in the wing pattern being paler gray and a larger size, but the male genitalia of both subspecies are almost identical. The female genitalia of *E. languidata incrassata*, represented by a single specimen only, exhibit slight differences from those of the nominate subspecies, such as the size and shape of the lamella postvaginalis. The results of the molecular studies show that the genetic distance between them is 0.5–0.9% (see Table 2).

**Table 2.** Genetic distances among eleven *Euryobeidia* samples and two outgroups based on COI barcodes.

	Voucher/ Sample ID	Species name	1	2	3	4	5	6	7	8	9	10	11	12	13
1	CRICATAS00283	*E. languidata languidata*													
2	CRICATAS00220	*E. languidata incrassata*	0.005												
3	CRICATAS00222	*E. languidata incrassata*	0.009	0.005											
4	ARB00027908	*E. xuei*	0.036	0.036	0.038										
5	IOZ LEP M 52355	*E. ellipsoidea*	0.053	0.056	0.058	0.030									
6	CRICATAS00247	*E. tigratoides tigratoides*	0.044	0.044	0.049	0.030	0.044								
7	CRICATAS00239	*E. tigratoides tigratoides*	0.044	0.044	0.049	0.030	0.044	0.000							
8	IOZ LEP M 22920	*E. tigratoides leopardiformis*	0.043	0.043	0.047	0.027	0.038	0.009	0.009						
9	CRICATAS00276	*E. largeteaui*	0.059	0.058	0.059	0.052	0.070	0.056	0.056	0.054					
10	CRICATAS00263	*E. largeteaui*	0.059	0.058	0.059	0.052	0.070	0.056	0.056	0.054	0.000				
11	CRICATAS00250	*E. quadrata*	0.055	0.058	0.059	0.046	0.056	0.046	0.046	0.041	0.067	0.067			
12	CCDB-11874-H01	*Eurychoria flavirupta*	0.056	0.056	0.059	0.053	0.062	0.052	0.052	0.052	0.073	0.073	0.056		
13	CRICATAS00211	*Epobeidia tigrata*	0.097	0.097	0.099	0.091	0.103	0.097	0.097	0.093	0.102	0.102	0.096	0.084	

##### Redescription.

Forewing length 24.9–28.0 mm in males, 26.5 mm in the single female (19–23 mm in *E. languidata languidata*). Ground color grayish-white, wings on upper- and underside covered with numerous pale gray spots and streaks (more numerous, larger, dark gray spots in *E. l. languidata*). More detailed general characters see the generic description. ***Head*.** Antennae filiform in both sexes, laterally with a pair of long, spine-like setae on each segment. Frons pale yellow, with a small, rounded protrusion in the middle near the base. Labial palpus pale yellow, slightly extending beyond frons. Vertex covered with erect, pale yellow scales. Chaetosemata weak. Proboscis short. ***Thorax*.** Dorsum pale yellow, two large separate black dots on mesothorax. Patagia and tegulae pale yellow, a small black dot at center of each patagium, a small black dot at base and a moderately large black dot at middle of each tegula. Legs pale yellow with a few dark spots. Index of spurs 0-2-4. Hind tibia not dilated, without scent-brush in males. Forewing not elongate, rather broadly triangular and arched at basal part of costa, apex angled, termen smoothly curved. Fovea absent. Forewing grayish-white, covered with lots of dark gray spots or streaks, especially on wing base, costa, inner margin and outer margin; antemedial line appearing as three dark gray spots on the upper and lower veins of cell and 2A respectively; six dark gray spots present outside the discal spot, positioned on veins R, M_1_, M_3_, CuA_1_, CuA_2_, and 2A, the first two spots commonly fused, the second two spots on M_3_ and CuA_1_, located further outwards, almost fused with the distal band; discal spot large, dark gray, elongate-oval, weakly curved inwards; terminal area with a band of tiny dark gray streaks, getting broader and more dense towards apex; fringes dark gray. Hindwing grayish-white, tinged with dark gray and pale yellow terminally, yellow part mixed with short gray transverse streaks; postmedial line similar to that of forewing, but spots smaller; discal spot present as a small dark gray dot; terminal line appearing as a row of black spots at the end of veins; fringes black corresponding to those spots, pale yellow between veins. Underside almost identical to upperside, but paler. ***Pregenital abdomen*.** Abdomen yellow, dorsal side lightly grayish-yellow, with dark gray spots at middle of each segment (a pair of two spots on first tergite which are fused in the middle on second tergite and get gradually more fused and reduced in size until tergite eight); lateral side with a row of black spots, and ventral side scattered with irregular dark gray spots. Tergites and sternites not conspicuously modified. The 8^th^ segment in males slightly elongate, broader than the 7^th^ segment, posteriorly slightly sclerotized, posterior edge straight. Tympanal organs of moderate size, without lacinia. Sterno-tympanal process, setal comb, and coremata absent.

***Male genitalia*.** Uncus moderately long, “bird-headed”, dorsal apical portion distinctly expanded semicircularly, densely covered with setae, “beak” portion small, pointed in lateral view, stem portion slightly curved, basal portion weak. Socii small, with fine setae. Gnathos weak, with short, narrow, sclerotized lateral arms which are separated by a large (membranous) opening at middle. Tegumen large, with long, stout lateral arms, distally swollen, curved ventrad. Transtilla long, partly broadened, ribbon-like, slightly sclerotized. Valva slender, apex strongly curved dorsad, with a small, faintly visible notch more ventrally. Costa narrow, smooth, apex slightly protruding. Cucullus well developed, from apex reaching back to center of valva. Basal saccular process broad, lamellar, dorsal edge serrate. Juxta broad, heavily sclerotized, groove-like. Saccus rounded. Aedeagus slender, curved, apically tapering, with an arrow-shaped tip in lateral view. Cornuti consisting of multiple irregularly joined sclerotized folds and patches.

***Female genitalia*.** Ovipositor rather short, papillae anales small, densely setose. Apophyses anteriores short, about ½ length of the very thin apophyses posteriores, basal two thirds of the former strongly broadened. A broad, triangular sclerite with a small tip present between the bases of apophyses posteriores. Lamella antevaginalis well developed, with many large irregular serrations. The ventral central plate of lamella postvaginalis strongly sclerotized, large, I-shaped; dorsal layer less sclerotized. Introitus bursae placed asymmetrically on left side, strongly sclerotized. Posterior part of bursa strongly sclerotized, barely twisted, with a few spines on left side, proximally swollen close to the bursa copulatrix, distally tapering, with a small colliculum-like structure adjacent to ductus seminalis. Anterior part of bursa almost oval, the distal ¾ with many spines inside and the proximal ¼ membranous, without spines.

##### Distribution.

China (Hainan).

##### Remarks.

This subspecies was originally described as *Euryobeidia incrassata* by Xiang et al. (2017). In their paper, the adult holotype, and the male genitalia of a paratype were illustrated, with the treatment of the new species primarily based on the distinctive genitalia of the latter, which set it apart from all other species in the genus. However, a detailed examination of the male genitalia of the holotype, two paratypes and non-types revealed that they were almost identical with those of *E. languidata languidata* (Walker, 1862) and *incrassata* therefore should be treated as a distinct subspecies of the latter. The only paratype of *E. incrassata* with distinctive male genitalia is redescribed in the present study as a new species, *E. supercostata* sp. nov. In addition, the authors examined all remaining paratypes and all freshly collected non-type males of *E. languidata incrassata* stat. nov. by dissecting the genitalia or brushing off scales to reveal the uncus and the characteristic part of the valva, ensuring that they were not confused with the almost identical in appearance and sympatric *E. supercostata* sp. nov.

#### ﻿ Euryobeidia supercostata

Taxon classificationAnimaliaLepidopteraGeometridae

Liu, Stüning & Han sp. nov.

73973B9E-F01E-5F50-80A5-BE7A3C2CE449

https://zoobank.org/81F55739-D56C-4F66-94F9-AC9E7E12B56B

Figs 14, 15, 44

Euryobeidia incrassata Xiang & Han, 2017, Zootaxa 4317 (2): 372 (part).

##### Type material.

***Holotype*.** China – **Hainan Province** • ♂; Lingshui, Diaoluoshan; 8 May 1984; coll. Gu Maobin; IOZ-CAS slide no. Geom-4140; IZCAS.

##### Diagnosis.

This new species is nearly identical in habitus, pattern, and coloration to the sympatric Euryobeidia languidata incrassata Xiang & Han, but it is considerably smaller in size. Moreover, there are some extremely subtle differences. For example, the section near the inner margin of the forewing has almost no black streaks; the hindwing discal spot is very small, appearing as a tiny dot. However, with only one specimen, we cannot be certain if these are stable characters. In addition, the male genitalia of this species, featuring an elongated uncus and an extremely broad costal protrusion, are distinctly different from those of other congeneric species.

##### Description.

The length of the forewing in the single male is ~20 mm. The wing pattern is almost identical to that of ssp. incrassata (see the redescription of *E. languidata incrassata*), except for the uncertain subtle differences already mentioned in the diagnosis.

***Male genitalia*.** Uncus long, subapical part (or stem) conspicuously elongated, slightly dilated dorsally, densely covered with setae, beak-like part long and stout. Socii absent or barely visible (the single type specimen). Gnathos weak, with a pair of short, fine sclerotized lateral arms, widely open or membranous in the middle. Tegumen moderately short (compared to most of other members of the genus), distally strongly curved ventrad. Transtilla long, broad, ribbon-like, slightly sclerotized. Valva broad, apex less curved dorsad (compared to most of congeneric species), ventral margin smooth, without notch. Costa with a markedly broad projection close to apex, dorsal edge faintly serrated in the middle. Cucullus well developed, from middle to apex of valva. Basal saccular process broad, lamellar, dorsal edge with minute denticles. Juxta strongly sclerotized, groove-like. Saccus short, rounded. Aedeagus slender, apically slightly tapering. Cornuti consisting of multiple irregularly joined sclerotized folds and patches.

**Female.** Unknown.

##### Distribution.

China (Hainan).

##### Etymology.

The specific name is derived from the extremely broad costa of the male genitalia.

##### Remarks.

The holotype of *Euryobeidia supercostata* sp. nov. was originally designated as a paratype of *E. incrassata* Xiang & Han, 2017. However, our examination of the holotype revealed that *E. incrassata* should be treated as a distinct subspecies of *E. languidata* (see *E. languidata incrassata* stat. nov.).

#### ﻿ Euryobeidia yakushimensis

Taxon classificationAnimaliaLepidopteraGeometridae

Inoue, 1976 stat. nov.

E77EDDEA-299E-5505-939A-48BB72826139

Figs 16, 17, 42, 43, 51, 52

Euryobeidia languidata yakushimensis Inoue, 1976, Tinea 10(2): 20. Holotype, Yakushima Isl., S. Japan (NHMUK) [images examined].

##### Material examined.

Japan – **Kagoshima Pref.** • 1 ♂; Yakushima Island, Miyanoura, Siratani; alt. 600 m; 18–20 Jun. 1993; M. Owada leg.; gen. prep. no. RS7496; CRS/ NIAES (shown by Sato 2011: pl. 1-015-12) • 1 ♀; Yakushima Island, Mt. Toimodake; alt. 1016 m; 26 Jun. 2008; Y. Kubota leg.; gen. prep. no. RS8879; CRS/ NIAES (shown by Sato 2011: pl. 1-015-13) • 1 ♂; Yakushima Island, Yakusugi Land; alt. 1000–1300 m; 12 Jul. 2008; Y. Kubota leg.; gen. prep. no. RS7411; CRS/ NIAES • 1 ♂; same locality and collector as for preceding; 16 Aug. 2009; gen. prep. no. RS7407; CRS/ NIAES • 1 ♀; same locality and collector as for preceding; 5 Jul. 2008; gen. prep. no. RS7412; CRS/ NIAES • 1 ♀; same locality and collector as for preceding; 5 Jul. 2009; gen. prep. no. RS7808; CRS/ NIAES.

##### Diagnosis.

This species is the only one with an almost unspotted wing pattern in the genus *Euryobeidia*, making it very easy to distinguish from other congeneric species. In terms of genitalia, this species is quite similar to *E. languidata*, indicating that they are rather closely related. However, there are still many stable differences between them, and they can be easily distinguished by the following characters: 1. Apex of the uncus is hardly dilated in *E. yakushimensis*, whereas it is distinctly inflated in *E. languidata*; 2. Costa of the male genitalia, with a small process in the middle in *E. yakushimensis*, while it is smooth, without process in *E. languidata*; 3. The triangular sclerite between the bases of the apophyses posteriores is distinctly narrower in *E. yakushimensis* than in *E. languidata*; 4. Lamella postvaginalis is smaller in *E. yakushimensis* than in *E. languidata*; 5. The spines on the posterior part of the bursa are sparser in *E. yakushimensis* than in *E. languidata*.

##### Redescription.

Wingspan 35–41 mm in males, 40–42 mm in females. Forewing almost entirely covered with large dark gray patches, the grayish-white ground color remaining only in narrow bands along the position of postmedial line or forming a few small, patches on discal cell or antemedial line. Hindwing similar to forewing, with a yellow marginal band intermingled with several small, dark gray transverse streaks and spots. Underside almost identical to upperside, but slightly darker. ***Male and female genitalia*.** The morphology of the genitalia is quite similar to that of *Euryobeidia languidata* (see description of *E. languidata incrassata*), with the following different features: Uncus fine, stick-like, apex rounded, bent ventrad, dorsal apical portion barely dilated. Apical lobe-like protrusion and ventral notch of valva are more conspicuous. Costa exhibits a distinctive small protruding structure in the center. The triangular sclerite between the bases of the apophyses posteriores is narrower and the lamella postvaginalis is smaller compared to *E. languidata*. Basal part of apophyses anteriores weakly broadened. The spines on the posterior part of bursa are sparser than in *E. languidata*. Anterior part of bursa almost oval, the upper ⅔ with numerous spines inside and the lower ⅓ membranous (the membranous part is smaller in *E. languidata*, but probably variable in size).

##### Distribution.

Japan (Yakushima).

##### Remarks.

Sato (2011) proposed that *E. languidata yakushimensis* should be treated as a distinct species based on his comparative study of *E. languidata languidata* and *E. languidata yakushimensis* from Japan. In the present study, we additionally include specimens from continental China and Vietnam, as well as the new subspecies *E. languidata incrassata* from Hainan, in our comparative analysis. The results show that in addition to significant differences in wing pattern, there are also specific differences in male and female genitalia (see the previous diagnosis and description). We agree with Sato’s comments on *E. languidata yakushimensis* and elevate it to species rank.

#### ﻿ Euryobeidia ellipsoidea

Taxon classificationAnimaliaLepidopteraGeometridae

Xiang & Han, 2017

93CBC23C-983C-5630-B5FB-C8269D78242E

Figs 9, 18

Euryobeidia ellipsoidea Xiang & Han, 2017, Zootaxa 4317 (2): 372.

##### Type material examined.

For figures of the male holotype and a female paratype, their genitalia and detailed type material data from IZCAS and ZFMK, see Xiang et al. (2017).

##### Additional material examined.

China – **Sichuan Province** • 1 ♀; “Chasseurs indigènes de Tâ-tsien-lou, Récolte de 1910”; Kangding; 1910; local collectors; coll. Charles Oberthür, ex coll. Wehrli, ZFMK.

##### Diagnosis.

This species is shown for comparison with *Euryobeidia xuei* sp. nov. Diagnostic characters are provided under *E. xuei* sp. nov. A female paratype in excellent condition deposited in the ZFMK collection is figured here for the first time (Fig. 18). Moreover, an additional female of larger size and rather untypical pattern is shown (see Fig. 9), discovered under ZFMK material during the present study (identified by genitalia dissection, gen. prep. no. 2459-DS).

##### Distribution.

China (Gansu, Sichuan, Shaanxi).

#### ﻿ Euryobeidia xuei

Taxon classificationAnimaliaLepidopteraGeometridae

Liu, Stüning & Han sp. nov.

CE6E62A3-0DA4-5242-8B06-7634DCE4542A

https://zoobank.org/B08EF2BA-5D19-4BB8-A849-CEC57D1FB86C

Figs 19, 45, 58

##### Type material.

***Holotype*.** Vietnam – • ♂; North Vietnam, Cha-pa, Mt. Fan-si-pan; 22°15'N, 103°46'E; 1500–1800 m; 10 Jun.–6 Jul. 1994; leg. V. Sinjaev & einh. Sammler; lux (light trap); gen. prep. no. 2458-DS; ZFMK. ***Paratype*.** Vietnam – • 1 ♂; same collection data as for holotype; ZFMK.

##### Additional material examined.

China – **Yunnan Province** • 1 ♂; Yuxi, Zhenyuan, Qianjiazhai, Ailaoshan; 24.277°N, 101.264°E; 2200 m; 5 Aug. 2011; Kitching & Ashton leg.; KIZCAS, ARB00027908 • 1 ♂ or ♀ (image only); Diqing, Weixi; 27.358805°N, 98.965343°E; 2720 m; 01 Aug. 2024; Photographed by Fan Gao.

##### Diagnosis.

*Euryobeidia xuei* is generally similar to *E. languidata* (Walker, 1862) in habitus, pattern, coloration and size, but can be distinguished from the latter on the basis of the shorter, less elongate wings, with apex and tornus more rounded and the compact, partly fused, very dark basal spotting, and a large, nearly fully fused, black discal spot forming a triangular shape in forewing which renders it more similar to *E. ellipsoidea* Xiang & Han, 2017. It differs from the latter and also from *languidata* by the complete or almost complete absence of a large spot or a dense group of smaller spots in the middle of the hind margin of the forewing. This feature that rarely occurs in other species and the extreme reduction of spots in the hindwings are distinctive for *E. xuei*.

The structure of the male genitalia is quite similar to that of *E. ellipsoidea*, especially the shape of valva and the presence of a large, plate-like structure of the uncus, which indicates a rather close relationship of the two taxa. Distinguishing characters are the round, apical lobes of the valve costa which is markedly larger in *xuei* and the stalk connecting the central, beak-shaped part of uncus and the large dorsal plate is longer and narrower, the plate itself has a different shape. Moreover, the 3% genetic distance based on molecular data separates this taxon from *E. ellipsoidea*, justifying its recognition as a distinct, but closely related species.

##### Description.

Forewing length 19–21 mm in males. Similar to *E. languidata*, but wing-shape different: wings shorter, broader, apex and tornus of forewing and hindwing more rounded.

***Head*.** Antennae filiform, agreeing with generic description, shaft dorsally covered with silvery-gray scales. Labial palpus dark gray, with a few yellow scales at base of first segment, the latter with moderately long, obliquely upright scales. Second joint rather smooth, third joint very small, tapering. Frons narrow, covered with dark brownish-gray scales, those on dorsal ½ smooth, slightly elongated, basal ½ with a brush of more strongly elongated scales, covering a small, rounded protrusion of the integument. Vertex small, consisting of large, dark gray, obliquely upright scales, forming a triangle. Posterior of it and around the head runs a conspicuous, yellow collar. Chaetosemata present, near eye-margin, consisting of a few sensillae only. ***Thorax*.** Dorsum as described in the generic description, anteriorly yellow, posteriorly grayish yellow, with two very large, almost black spots behind each other. Patagia yellow with large, dark gray spots, tegulae with a yellow transverse band, a basal dark gray spot and a second spot and lighter gray hair-scales posteriorly. Legs yellow and yellowish gray, with some black dots. Tibia of hind-leg not dilated, without scent pencil. Wings with ground color clear white. Pattern of very dark, rather large, round spots, compact and partly fused near base of forewing. The more internal dots are suffused with brown scales, while the large apical patch and narrow bands along costa and anterior ⅔ of termen are dark silvery gray. A large spot in the middle of the hind margin absent, resulting in a broad, white band stretching obliquely through the forewing from near apex to hind margin. This seems to be the most obvious distinguishing element and is present in all known specimens. There are small yellow streaks near tornus. Fringe almost entirely black. Spots on hindwing reduced in size or absent, rendering the white area the largest of all species. Discal dot round, rather large. Base of hindwing with a group of very small dots. Yellow band along hindwing termen narrow, only sparsely dotted, fringe yellow with black, almost round dots. Underside with pattern very similar, but much paler. Venation agreeing with generic description, except that CuP is represented by a very faint fold, without a weakly sclerotized basal portion. ***Pregenital abdomen*.** Ground color of proximal ¾ of dorsal side pale yellowish gray, distal ¼ deeply yellow. All tergites with spots or patches of different sizes, shapes, and colors: T1 pale gray, with a deep incision proximally in the middle. Spots from T2 to T8 almost black. T2, T3 smaller, almost semicircular, T4-T6 rectangular, transverse bands (T6 smaller), T7 a very small spot, T8 a pair of rather large, quadrate patches (see Figs 19, 58). Laterally a row of black, irregular spots, decreasing in size towards tip of abdomen. Lateral and ventral sides deep yellow, with moderately elongated scales and irregularly distributed black spots of different size and shape. Shape and number of these spots is due to ample variation. Integument without distinct variations, tergite 8 narrowly rectangular, sternite 8 much larger, laterally and distally rounded. Tympanal organs of moderate size, shallow, without lacinia. Setal comb on sternite 3 and sterno-tympanal process absent. Tergal phragma T1/T2 long, strap-like.

***Male genitalia*.** Uncus with a central, beak-shaped part, carried by a long and narrow “neck” which combines it with the forked base and the semicircular connection with the tegumen. From the central part, a long and narrow, spined stalk arises which supports a large plate of unknown function. It is rounded posteriorly and tapering to a slightly narrower end anteriorly (Fig. 45). The gnathos consists of tiny, sclerotized lateral arms, which are widely separated from each other. Valves long and narrow, with the costa smooth, slightly protruding on both sides. Apex of valva strongly curved back, with a large, round, transparent, sclerotized plate (but hardly visible). Sacculus with a large, oval, basal plate, its margin irregularly indented and covered with minute spines. Aedeagus moderately long, the sclerotized distal ½ flat and terminating with a short spine at the rather broad apex. Vesica with two cornuti of different shape which are fused at one end.

**Female.** Unknown.

##### Distribution.

Vietnam, China (Yunnan).

##### Etymology.

The specific epithet is honoring the renowned Chinese taxonomist Prof. Dayong Xue for his outstanding contributions to the study of Geometridae.

#### ﻿ Euryobeidia largeteaui

Taxon classificationAnimaliaLepidopteraGeometridae

(Oberthür, 1884)

3067910A-681D-5517-96EB-DC636FA95F71

Figs 20–24, 59

Rhyparia largeteaui Oberthür, 1884, Études ent. 10: 32, pl. 1, fig. 5. holotype ♂, China: Kouy-Tchéou, in coll. ZFMK.
Euryobeidia largeteaui : Wehrli 1939, in Seitz, Gross-Schmett. Erde 4 (Suppl.): 269. Unavailable.
Euryobeidia largeteaui : Fletcher 1979, The Generic Names of Moths of the World 3: 84; Xiang et al. 2017, Zootaxa 4317 (2): 373.

##### Type material examined.

**Figures 20–37.**
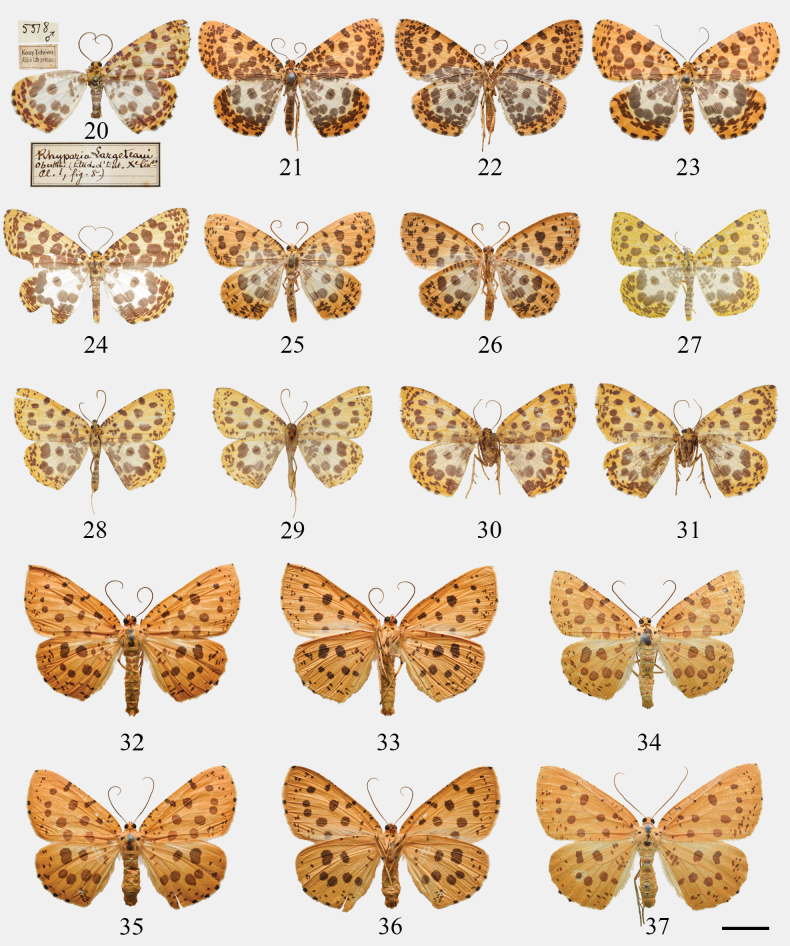
Adults of *Euryobeidia* species. **20–24.***E. largeteaui* (Oberthür): **20.** The holotype of *Rhyparia largeteaui* Oberthür, male, Guizhou, China, ZFMK; **21.** Male, Hunan, China, upperside, CRICATAS; **22.** Ditto, underside; **23.** Female, Hunan, China, CRICATAS; **24.** Male, N. Vietnam, CKY; **25.***E. quadrata* Xiang & Han, male, Hunan, China, upperside, CRICATAS; **26.** Ditto, underside; **27–31.***E. tigratoides leopardiformis* subsp. nov.: **27.** Holotype, male, Sichuan, China, IZCAS; **28.** Paratype, male, W. Hubei, China, upperside, ZFMK; **29.** Ditto, underside; **30.** Paratype, female, W. Hubei, China, upperside, ZFMK; **31.** Ditto, underside; **32–37.***E. tigratoides* sp. nov., Hainan Is., China, CRICATAS/ IZCAS: **32.** Holotype, male, upperside; **33.** Ditto, underside; **34.** Paratype, male; **35.** Paratype, female, upperside; **36.** Ditto, underside; **37.** Paratype, female. Scale bar: 10 mm.

***Holotype*.** China – **Guizhou Province** • ♂; Kouy-Tchéou (Guizhou); Abbé Largeteau leg.; gen. slide Wehrli no. 5578; ex coll. Oberthür, ex coll. Wehrli, ZFMK. (left forewing absent; see Fig. 20)

##### Additional material examined.

China – **Hunan Province** • 20 ♂♂, 12 ♀♀; Huaihua City, Xupu County, Taojinping Township, Shannaoao Village, Mountain woodland; 800 m; Jul. 2022; Chao Dai leg.; GenBank nos: PQ083536, PQ083541; CRICATAS, CRICATAS00251 to CRICATAS00282. – **Hubei Province** • 4 ♂♂, 1 ♀; W. Hubei Province, Wufeng, Yizhuxiang Mt.; 1560 m; Jun. 1998; Wang & Li leg.; ZFMK. Further 26 ♂♂♀♀ in ZFMK from Zhejiang, Hunan, Fujian, Guangdong, mainly ex coll. Wehrli, Oberthür, Höne. Vietnam – • 1 ♂; N. Vietnam, Lao Cai, Sa Pa; 1500 m; 25–28 May 1997; B. Tanak leg.; CKY • 6 ♂♂; N. Vietnam, Cha-pa, Mt. Fan-Si-Pan; 22°15'N, 103°46'E; 1600–1800 m; leg. Sinjaev & Simonov; ZFMK.

##### Diagnosis.

This widespread and abundant species is presented here for comparison with *E. tigratoides leopardiformis* subsp. nov., due to their nearly identical appearance. Diagnostic characters are given under the description of the latter.

##### Distribution.

China (Gansu, Zhejiang, Hubei, Jiangxi, Hunan, Fujian, Taiwan, Guangdong, Hong Kong, Guangxi, Sichuan, Chongqing, Guizhou), Vietnam.

##### Remarks.

A specimen from Baoxing, Sichuan, China, identified by Xiang et al. (2017) as *E. largeteaui*, is described in the present study as a new subspecies of *E. tigratoides* sp. nov., and is designated as the holotype.

#### ﻿ Euryobeidia quadrata

Taxon classificationAnimaliaLepidopteraGeometridae

Xiang & Han, 2017

02884497-086B-5E3C-8531-1167F1542E62

Figs 25, 26, 60, 61

Euryobeidia quadrata Xiang & Han, 2017, Zootaxa 4317 (2): 374.

##### Type material examined.

The description was based on a large series of male and female specimens, the holotype designated from Zhejiang. Detailed type material data from IZCAS and ZFMK, see Xiang et al. (2017).

##### Additional material examined.

China – **Hunan Province** • 1 ♂; Huaihua City, Xupu County, Taojinping Township, Shannaoao Village, Mountain woodland; 800 m; Jul. 2022; Chao Dai leg.; GenBank no.: PQ083535; CRICATAS, CRICATAS00250.

##### Diagnosis.

This species is also shown for comparison with *E. tigratoides* sp. nov. and *E. tigratoides leopardiformis* subsp. nov., particularly the latter. Diagnostic characters are provided under *E. tigratoides leopardiformis* subsp. nov.

##### Distribution.

China (Anhui, Zhejiang, Hubei, Hunan, Jiangxi, Fujian, Guangdong, Hong Kong, Guangxi, Sichuan).

#### ﻿ Euryobeidia tigratoides

Taxon classificationAnimaliaLepidopteraGeometridae

Liu, Stüning & Han sp. nov.

33976CE5-A171-5DD9-B8A8-8655DB644BD7

https://zoobank.org/9BDF3320-09C3-45E7-8629-9BF41FE1D955

Figs 32–37, 46, 47, 54, 63, 64

##### Type material.

***Holotype*.** China – **Hainan Province** • ♂; Lingshui, Diaoluoshan; 922 m; 01–03 Apr. 2024; Bo Liu, Wei Lin & Miaofeng Xu leg.; CRICATAS/ IZCAS, CRICATAS00230. ***Paratypes*** (19 ♂♂, 6 ♀♀). China – **Hainan Province** • 2 ♂♂, 3 ♀♀; same locality as for holotype; 20 Apr. 2023; Bo Liu leg.; gen. prep. nos. CRICATAS00243, CRICATAS00244; CRICATAS/ IZCAS/ ZFMK • 2 ♂♂; same locality as for holotype; 10 May 2023; Bo Liu leg.; GenBank no. PQ083534; gen. prep. no. CRICATAS00247; CRICATAS/ IZCAS • 7 ♂♂, 1 ♀; same collection data as for holotype • 7 ♂♂; same locality as for holotype; 07–12 May 2024; Bo Liu & Wei Yan leg.; GenBank no.: PQ083538; CRICATAS, IZCAS /ZFMK • 1 ♂; Lingshui, Diaoluoshan; 997 m; 21 Apr. 2025; Bo Liu & Wei Yan leg.; CRICATAS • 2 ♀; Lingshui, Diaoluoshan; 974 m; 10 Jun. 2025; Bo Liu & Wei Yan leg.; CRICATAS.

**Figures 38–43.**
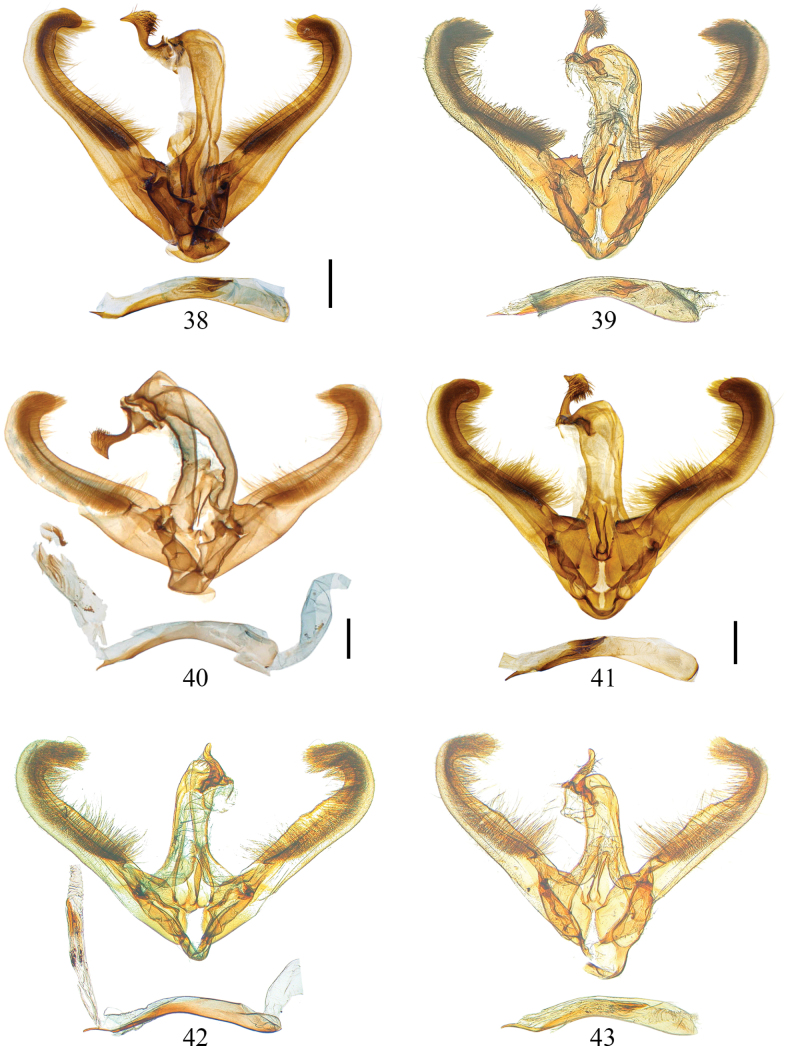
Male genitalia of *Euryobeidia* species. **38, 39.***E. languidata* (Walker): **38.** Fujian, China, gen. prep. no. CRICATAS00283; **39.** Tokyo, Japan, gen. prep. no. RS8877; **40, 41.***Euryobeidia languidata incrassata* Xiang & Han, stat. nov., Hainan Is., China: **40.** The holotype of *E. incrassata* Xiang & Han, IOZ-CAS slide no. Geom-7235; **41.** Non-type, gen. prep. no. CRICATAS00220; **42, 43.***E. yakushimensis* Inoue, stat. nov., Yakushima Is., Japan, gen. prep. nos. RS7407, RS7411. Scale bars: 1 mm.

##### Diagnosis.

*Euryobeidia tigratoides* is the only entirely orange/ yellow species without a white pattern or area on the hindwing. This external character, together with its large body size, makes it very easy to distinguish from all other congeners. In addition to its distinct appearance, this species can also be readily differentiated from all other congeneric species (except for the next new subspecies to be described: *E. tigratoides leopardiformis* subsp. nov.) by the following two features of the male genitalia: 1.There is a protruding, rounded lobe bare of setae at the apex of valva, with a neighboring evident notch in *E. tigratoides*, while in other species, it is absent or inconspicuous. 2. The basal process of the sacculus is broader than in any other congeneric species.

##### Description.

Forewing length 21.5–24.4 mm in males, 25.2–26.1 mm in females. Ground color orange or yellow after fading, densely covered with numerous dark spots. More detailed general features see the previous generic description. ***Head*.** Antennae filiform in both sexes, ventro-laterally with a pair of long, spine-like setae on each segment. Frons narrow, covered with smooth, narrow, light orange scales, with a small, rounded, central protrusion near the base. Labial palpus slightly extending beyond frons. Vertex covered with erect, lamellar, light orange scales. Chaetosemata small, near eye margin. Proboscis short. ***Thorax*.** Dorsum bright orange, two large separate black dots present on mesothorax. Patagia and tegulae bright orange, patagium with a small black spot in most individuals, tegula with a small black spot at base and a large black spot at middle. Legs orange, a few small black spots mainly on the base, middle and end of the femur and tibia segments. Index of spurs 0-2-4. Hind tibia not dilated, without scent-brush in males. Wings entirely orange, without white pattern. Forewing not elongated, arched at basal part of costa, apex angled, termen smoothly curved, fovea absent. Forewing scattered with numerous small streaks or spots on basal, costal and terminal areas; antemedial line represented by three large dark spots, the large streaked patch on the middle of the costal aera, extending from the costa down to near the cell-fold (weak or barely visible in few individuals); six separate dark spots present outside the discal spot, getting larger from upper to lower; discal spot appearing as a large dark dot; fringes matching the ground color, interspersed with black. Hindwing scattered with numerous tiny spots or streaks on basal and terminal areas; the first two spots of postmedial line always fused, the second and third pairs are typically separate, the two spots within the second pair or within the third pair, very close or even connected only in few individuals; discal spot large, rounded. Underside of wings almost identical to upperside, but slightly darker. Area of wing-coupling pale. ***Pregenital abdomen*.** Abdomen covered with fine, orange scales; dorsal dark spots fewer than those of other congeners, and inconspicuous or absent in some individuals. Tergites and sternites not conspicuously modified. The 8^th^ segment in males slightly elongate, broader than the 7^th^ segment, posteriorly slightly sclerotized, posterior edge slightly concave in the center. Tympanal organs of moderate size, without lacinia. Sterno-tympanal process, setal comb and coremata absent.

***Male genitalia*.** Uncus short, “bird-headed”, dorsal apical part markedly dilated, densely covered with setae, “beak” part pointed in lateral view, stem short, strongly curved dorsad. Socii small, with fine setae. Gnathos weak, with a pair of short, fine sclerotized lateral arms only. Transtilla long, broad, sclerotized, band-shaped. Tegumen large, with long, stout lateral arms, distally significantly swollen, curved ventrad. Valva slender, apex strongly curved dorsad at a right angle, with a large, round, non-setose extension of costa, forming a conspicuous notch ventrally. Costa narrow, smooth. Cucullus well developed, from apex reaching back to center of valva. Basal process of sacculus quite broad, lamellar, dorsal edge with minute denticles. Juxta broad, heavily sclerotized, groove-like. Saccus small, rounded. Aedeagus slender, apically slightly tapering and ridge-like. Cornutus small, with a central groove. Bulbus ejaculatorius shorter than the aedeagus shaft, with a rather large cap.

***Female genitalia*.** Ovipositor very short, papillae anales small, densely setose. Apophyses anteriores slightly shorter than apophyses posteriores, basal ¼ slightly broadened. A narrow, triangular sclerite present between the bases of posterior apophyses. Lamella antevaginalis well developed, with large irregular serrations. The ventral central plate of lamella postvaginalis large, broad at top, narrow at bottom, apical center slightly concave; dorsal layer with a pair of large, sclerotized, irregularly shaped, lateral projections. Introitus bursae slightly displaced to left side, strongly sclerotized. Posterior part of bursa fine, strongly sclerotized and twisted, connected to the ductus seminalis at middle, proximally swollen close to the bursa copulatrix. Anterior part of bursa pyriform, the distal ½ with many spines inside, the proximal ½ membranous, without spines.

##### Distribution.

China (Hainan).

##### Etymology.

The specific name is derived from its potential mimicry model, the nominotypical subspecies of *Epobeidia tigrata* (Guenée).

#### ﻿ Euryobeidia tigratoides leopardiformis

Taxon classificationAnimaliaLepidopteraGeometridae

Liu, Stüning & Han subsp. nov.

AB238D9F-AEBB-5084-B2BF-5ABD3240BA44

https://zoobank.org/25680A5F-1A5D-421C-B916-AC92F486F9E5

Figs 27–31, 48, 49, 55

Euryobeidia largeteaui : Xiang et al. 2017, Zootaxa 4317 (2): 374 (part).

##### Type material.

***Holotype*.** China – **Sichuan Province** • ♂; Baoxing County, Dashuigou; 1591 m; 1–5 Aug. 2016; Le Cui leg.; gen. prep. no. Geom-04555; IZCAS, IOZ LEP M 22920. ***Paratypes*.** China – **Hubei Province** • 2 ♂♂, 1 ♀; W. Hubei Province, Wufeng, Yizhuxiang Mt.; 1560 m; Jun. 1998, Wang & Li leg.; gen. prep. nos. 2449-DS, 2450-DS; ZFMK.

**Figures 44–49.**
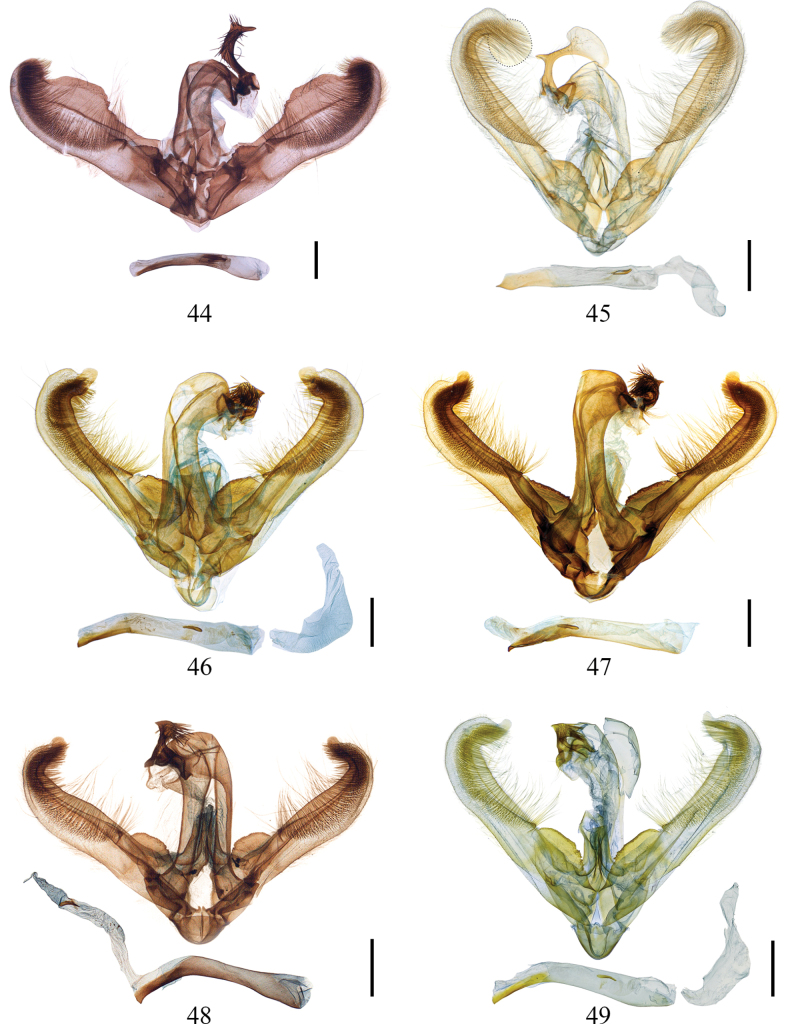
Male genitalia of *Euryobeidia* species. **44.***E. supercostata* sp. nov., holotype, Hainan, China, IOZ-CAS slide no. Geom-4140; **45.***E. xuei* sp. nov., holotype, male, Fansipan, Vietnam, gen. prep. no. 2458-DS; **46, 47.***E. tigratoides* sp. nov., paratypes, Hainan Is., China, gen. prep. nos. CRICATAS00247, CRICATAS00243; **48, 49.***E. tigratoides leopardiformis* subsp. nov.: **48.** Holotype, Sichuan, China, IOZ-CAS slide no. Geom-04555: **49.** Paratype, W. Hubei, China, gen. prep. no. 2449-DS. Scale bars: 1 mm.

**Figures 50–55.**
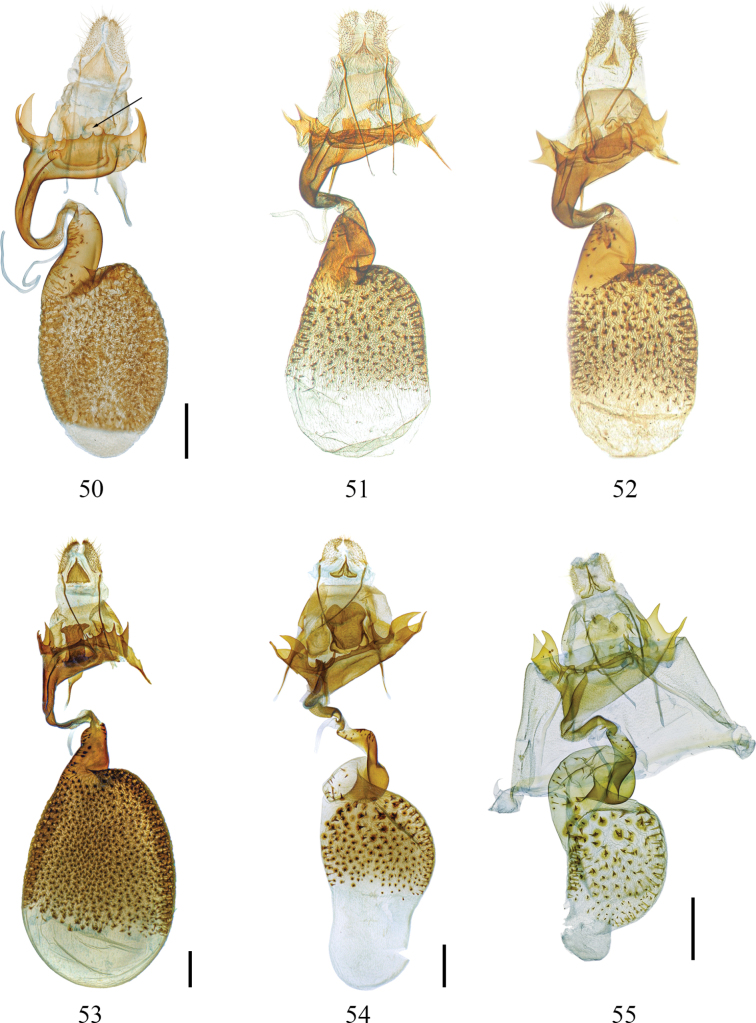
Female genitalia of *Euryobeidia* species. **50.***E. languidata* (Walker), Japan, gen. prep. no. 2460-DS; **51, 52.***E. languidata yakushimensis* Inoue, Yakushima Is., Japan, gen. prep. nos. RS7412, RS8879; **53.***Euryobeidia languidata incrassata* Xiang & Han, stat. nov., Hainan Is., China, gen. prep. no. CRICATAS00223; **54.***E. tigratoides* sp. nov., paratype, Hainan Is., China, gen. prep. no. CRICATAS00244; **55.***E. tigratoides leopardiformis* subsp. nov., paratype, W. Hubei, China, gen. prep. no. 2450-DS. Scale bars: 1 mm.

**Figures 56–64.**
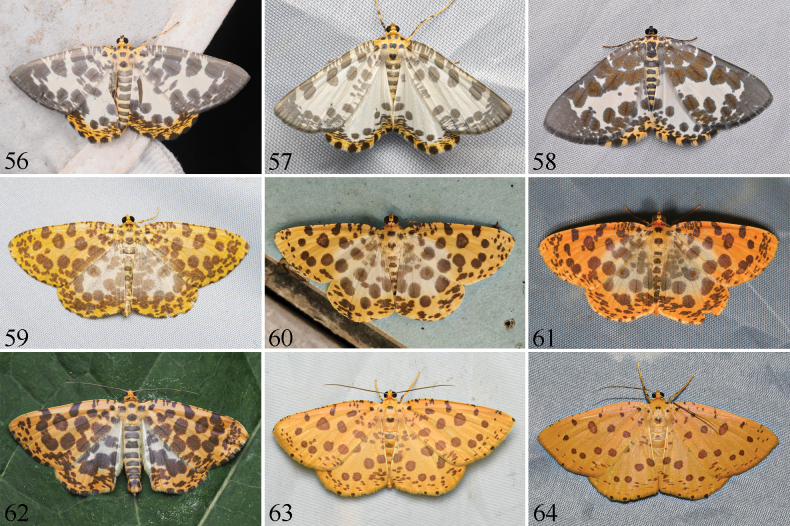
Living imagos of *Euryobeidia* species in resting position. **56.***E. languidata* (Walker), Fujian, China; **57.***E. languidata incrassata* Xiang & Han, stat. nov., Hainan, China; **58.***E. xuei* sp. nov., Yunnan, China; **59.***E. largeteaui* (Oberthür), Guizhou, China; **60.***E. quadrata* Xiang & Han, Hong Kong, China; **61.***E. quadrata* Xiang & Han, Anhui, China; **62.***E. tigratoides leopardiformis* subsp. nov. or *E. largeteaui*, Guangdong, China; **63.***E. tigratoides* sp. nov., male, Hainan, China; **64.***E. tigratoides* sp. nov., female, Hainan, China.

##### Diagnosis.

This new subspecies is significantly smaller than the nominate subspecies and exhibits a strikingly distinct appearance, characterized by the basal ⅔ of the hindwing being white. However, the highly similar male and female genitalia and a minimal genetic divergence of 0.94% (see Table 2) strongly indicate a close taxonomic relationship, thereby supporting the treatment of *leopardiformis* as a new subspecies of *E. tigratoides*. In addition to the markedly different wing patterns, the former can also be clearly distinguished from the latter by the following characters of male and female genitalia: the uncus is clearly longer with its stem straight or only slightly curved when observed from a lateral aspect, the spines on the posterior part of bursa are more concentrated compared to the nominate subspecies, and the lateral process of lamella postvaginalis is less sclerotized than in the nominate subspecies.

Furthermore, *Euryobeidia tigratoides leopardiformis* closely resembles the sympatric *E. largeteaui* (Oberthür) in size, habitus, coloration, and wing pattern, rendering them nearly indistinguishable by appearance alone. Another sympatric species, *E. quadrata* Xiang & Han, also similar in appearance to the two species mentioned above, can be distinguished by a combination of characters: a white patch or area on the forewing (this single feature is also present in some individuals of *E. largeteaui*) and the nearly fused second pair of dots of the postmedial line on the hindwing. The new subspecies can be readily differentiated from *E. largeteaui* and *E. quadrata* by the following genitalic characters: 1. Uncus is small, bird-headed, while in *E. largeteaui* it is large, semicircular; in *E. quadrata*, it is also larger, with an elongated apex and an extremely dilated, somewhat square, flake-like protrusion on the dorsal side. 2. Basal process of sacculus is quite broad with minute denticles along the dorsal edge, whereas in both *E. largeteaui* and *E. quadrata*, it is narrow and lacks denticles. 3. The valval apex bears a conspicuous lobe and a neighboring notch, which is absent or inconspicuous in the other two species. 4. Apex of aedeagus is moderately long, slightly tapering, and ridged; in contrast, it is rather long and rod-like in *E. largeteaui*, and short and broad in *E. quadrata*. 5. Lamella antevaginalis is well-developed with large, irregular serrations, whereas in *E. largeteaui*, it is large and triangular with an upright sclerite in the center, and in *E. quadrata*, consists of two semicircular sclerites. 6. Lamella postvaginalis is rather large and M-shaped, with a pair of large, slightly sclerosed lateral processes, whereas it is much smaller in *E. quadrata* and quite small, barely visible, in *E. largeteaui*.

##### Description.

Forewing length 18–20 mm in males, 21 mm in the single female. Adults of *E. tigratoides leopardiformis* are almost identical to *E. largeteaui* in habitus, coloration, pattern and size, there are no consistent features to distinguish them. Typically, the six spots of postmedial line are fused in pairs, with the second and third pairs sometimes also slightly fused in certain specimens. However, the wing pattern observed in all four known type specimens of *E. tigratoides leopardiformis* is also found in specimens with nearly identical patterns within the extensive *E. largeteaui* collection. Currently, we believe that the two taxa cannot be accurately distinguished based on adult external morphological characters other than genitalia.

***Male and female genitalia*.** The male and female genitalia are strikingly similar to those of *E. tigratoides tigratoides* (see the previous description of *E. tigratoides*), with the following stable differences: the shaft of the uncus is longer, its stem is less curved, and its dorsal apical part is less dilated; The spines on the posterior part of bursa are sparser; the lateral process of lamella postvaginalis is less sclerotized.

##### Distribution.

China (Sichuan, Hubei).

##### Etymology.

The subspecific name is derived from its potential mimicry model, the subspecies leopardaria (Oberthür) of *Epobeidia tigrata* (Guenée).

### ﻿Molecular analysis

**Figure 65.**
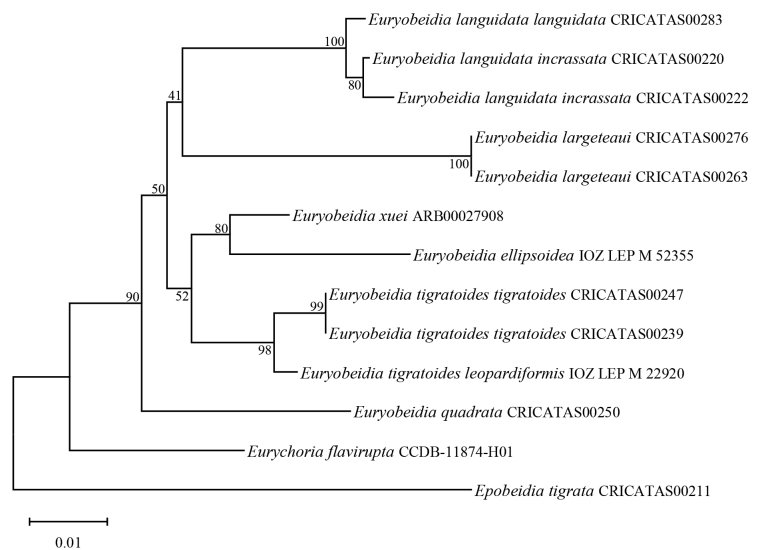
Neighbor-joining tree of the selected samples based on DNA barcode data.

Based on the currently available data, the following preliminary conclusions can be drawn. Interspecific genetic distances range from 2.7% to 7%, while intraspecific distances range from 0.5% to 0.9% (see Table 2). The neighbor-joining tree for *Euryobeidia* (Fig. 65) revealed that the two subspecies pairs (*E. languidata languidata*/ *E. languidata incrassata*, and *E. tigratoides tigratoides*/ *E. tigratoides leopardiformis*) each clustered tightly, supported by Bootstrap values of 100% and 98%, respectively. The genetic distance separating the members within the two subspecies pairs was only 0.5–0.9% for the former pair and 0.9% for the latter pair. This minimal separation strongly supports their classification as conspecific taxa.

A phylogenetic tree, constructed by BOLD Systems and based on sequences of the ‘100 nearest neighbors’, i.e., the species deemed most closely related to species of *Euryobeidia*, identified *Eurychoria flavirupta* Warren, 1903 (Papua New Guinea), also from the Baptini, as a potential close relative with a genetic distance of 5.2–7.3% (see Table 2) from species of *Euryobeidia*. However, *Eurychoria flavirupta* differs significantly from *Euryobeidia* in both external morphology and genitalic characters. The position of *Euryobeidia* within Baptini is still uncertain.

## ﻿Discussion

The genus *Euryobeidia* exhibits typical features of Baptini, such as vein R_2_ arising from the common stem of R_3-5_ on the forewing, the absence of a setal comb on sternite 3 of the male abdomen, and the set of male genitalia characters including a reduced gnathos, a broad, immaculate costal zone, often with a marginal process or angle, and a central zone of long setae of the valva. Stüning (2000: 110) pointed out in his article that the male genitalia of *Euryobeidia* reliably agree with the definition of the tribe Baptini given by Holloway [1994: 58], although showing some derived features, and confirmed that this genus should be placed within the Baptini. Xiang et al. (2017) also agreed that *Euryobeidia* fits well within Baptini based on their comparative studies. Species of *Euryobeidia* possess a distinctive suite of external morphological and genitalic structures (including, for example, an *Obeidia*-complex-like wing pattern, a highly modified and diverse uncus, and a more or less swollen tegumen in the male genitalia). These features set them clearly apart from other genera and form a separate group within the tribe Baptini. Consistently, molecular data also support this clear delimitation. The Neighbor-Joining tree, strongly supported by a Bootstrap value of 90% (Fig. 65), shows that the DNA barcoding results based on COI sequences from the present study place all *Euryobeidia* species within a single clade. The two unsequenced taxa (*E. supercostata* and *E. yakushimensis*) exhibit striking morphological similarity to the sequenced species. Due to this high congruence, it is hypothesized that their inclusion will not alter the current topology, thus preserving the monophyletic status of the genus *Euryobeidia*. Additionally, the most likely closely related genus available for study is *Eurychoria* Prout, 1916, which also belongs to the tribe Baptini. The genetic divergence between *Eurychoria flavirupta* and species of *Euryobeidia* (5.2–7.3%) is even smaller than that observed between some species within *Euryobeidia*, indicating a relatively close relationship between the two genera. However, *Eurychoria* species differ from *Euryobeidia* species in both external and genitalic characters, with the latter suggesting that the two may not in fact be the most closely related genera. The systematic position of *Euryobeidia* within Baptini remains uncertain. Furthermore, although Holloway (1994) and Pitkin (2002) conducted valuable initial studies on the Baptini tribe, significant gaps remain in our understanding of this group. Numerous issues persist, such as the lack of a strict morphological definition and unclear phylogenetic relationships among its members. Perhaps morphological studies of larvae or pupae combined with more comprehensive phylogenetic research on multiple members using multi-gene fragments or even whole genomes will better solve these problems in the future.

**Figure 66.**
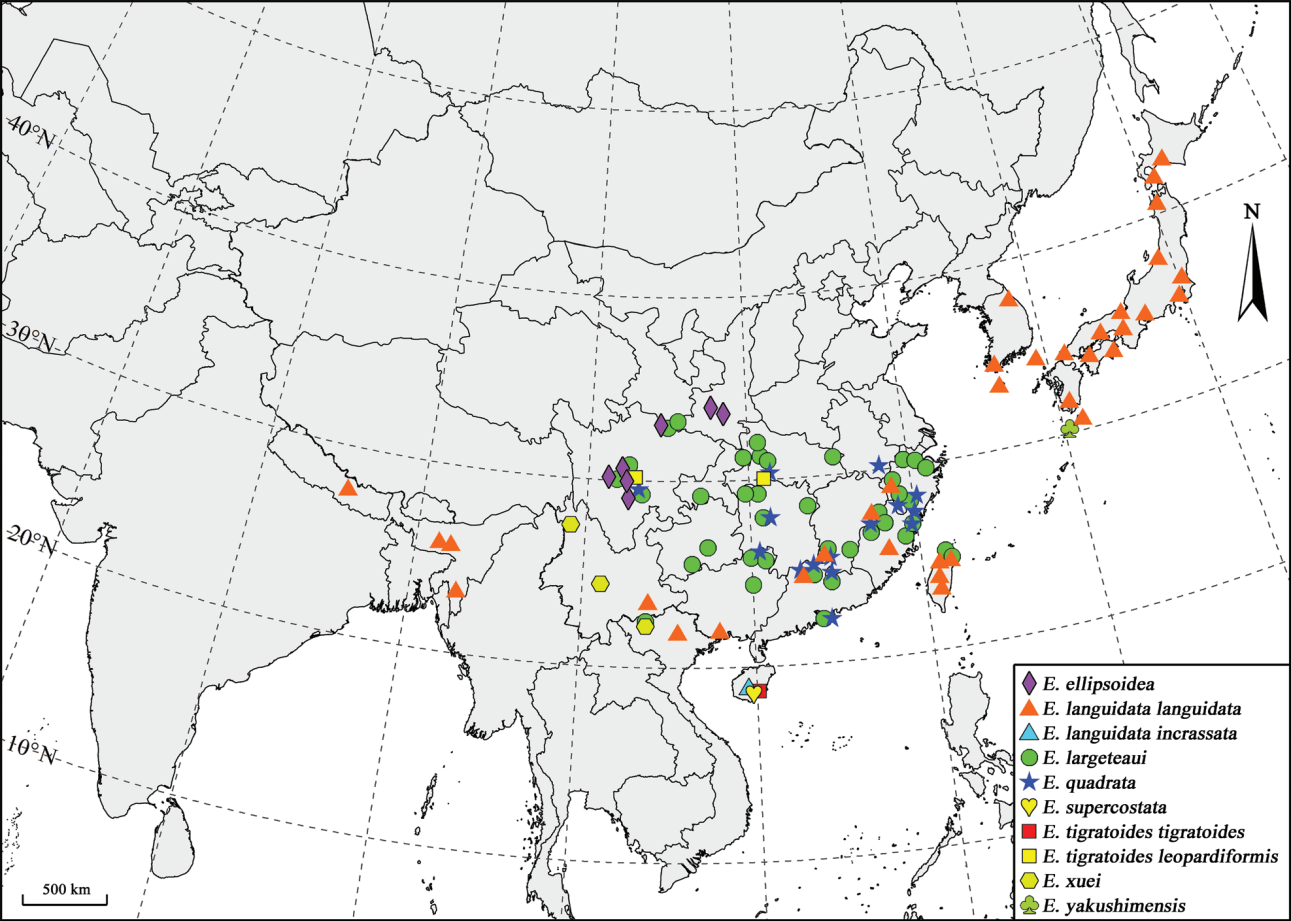
Distributions of *Euryobeidia* species. Notes: Distribution data are primarily derived from collection localities of examined specimens, with a limited number of distribution records obtained from iNaturalist (https://www.inaturalist.org) and literature (Sato 2011; Kim et al. 2016; Fu et al. 2020).

The genus *Euryobeidia* Fletcher, 1979 now comprises eight taxa at the rank of species and two additional taxa at the rank of subspecies, distributed in East, Southeast, and South Asia (Fig. 66). This genus exhibits exceptionally high species diversity in Southern China, encompassing all known species and subspecies of the genus except for *E. yakushimensis*. This level of diversity far surpasses that observed in other regions. This pattern strongly suggests that Southern China could be the center of origin or a radiating evolutionary center for this lineage. Furthermore, this high diversity might also be linked to the extremely high species and population diversity of its potential mimetic models (the *Obeidia*-complex and *Abraxas* Leach) within the same region.

As mentioned in the generic description, *Euryobeidia* species can be divided into two groups, based on their ground color: a grayish-white group that presumably mimics species of the large genus *Abraxas* and an orange/yellow group that possibly mimics species of the *Obeidia*-complex of genera, most certainly those of the genus *Epobeidia*. Notably, this grouping serves to describe morphological traits and interpret mimicry, as the coloration and patterns are likely shaped by mimicry rather than reflecting true relatedness. In fact, neither molecular analysis nor comparison of genitalic characters provides support for the two groups as natural phylogenetic lineages. The mimetic relationships between them will be described and illustrated in detail in a separate article in the future.

## Supplementary Material

XML Treatment for Euryobeidia

XML Treatment for Euryobeidia languidata

XML Treatment for Euryobeidia languidata incrassata

XML Treatment for Euryobeidia supercostata

XML Treatment for Euryobeidia yakushimensis

XML Treatment for Euryobeidia ellipsoidea

XML Treatment for Euryobeidia xuei

XML Treatment for Euryobeidia largeteaui

XML Treatment for Euryobeidia quadrata

XML Treatment for Euryobeidia tigratoides

XML Treatment for Euryobeidia tigratoides leopardiformis

## ﻿Additional information

### Use of AI

No use of AI was reported.

### Author ORCIDs

Bo Liu https://orcid.org/0009-0008-7003-4659

Dieter Stüning https://orcid.org/0000-0002-1748-4510

Hongxiang Han https://orcid.org/0000-0001-7538-2902

### Data availability

All of the data that support the findings of this study are available in the main text or Supplementary Information.

## Supplementary materials

Supplementary material 1 DNA sequences Data type txt https://binary.pensoft.net/file/1471992This dataset is made available under the Open Database License (http://opendatacommons.org/licenses/odbl/1.0/). The Open Database License (ODbL) is a license agreement intended to allow users to freely share, modify, and use this Dataset while maintaining this same freedom for others, provided that the original source and author(s) are credited.Bo Liu, Dieter Stüning, Hongxiang Han
